# Negotiating intersubjectivity by interpersonal and appraisal shifts in Chinese-English government press conference interpreting

**DOI:** 10.3389/fpsyg.2023.1106174

**Published:** 2023-03-01

**Authors:** Jun Xu, Yuxiao Liang

**Affiliations:** ^1^English Department, School of Foreign Languages, Hunan University, Changsha, Hunan, China; ^2^Department of Politics, Languages & International Studies, University of Bath, Bath, United Kingdom

**Keywords:** intersubjectivity, interpreting shift, interpersonal resources, government press conference, ideology

## Abstract

This study investigates how interpreting shifts of interpersonal and appraisal resources facilitated the successful negotiation of intersubjectivity at China's premier press conferences (PPCs) from 2016 to 2021. This study conducts a corpus-based critical discourse analysis of interpersonal shifts at the PPCs as defined by systematic functional linguistics. Quantitative results show that the interpreter is strongly inclined to utilize appraisal shifts which enhance (or soften) the positive (or negative) evaluations of the Chinese government in interpreting the journalists' questions and uses shifts to first-person plurals and inclinational modal verbs in interpreting the Chinese Premier's answers. Qualitative results show these shifts facilitate the direct or indirect reproduction of the government's official ideology (especially the notions of solidarity, change, resolution, and people's wellbeing) and the existing power relations between the government, media, and Chinese people (both authority and solidarity). It is concluded that the interpreter displays a strong tendency to use interpersonal shifts to ensure successful negotiation of intersubjectivity at the PPCs by ultimately reproducing the social *status quo*.

## 1. Introduction

The premier press conference (PPC), held live-streamed annually in March at the closing of China's “two sessions,” is a high-profile political and diplomatic discourse event, where the Chinese Premier answers around a dozen potentially challenging questions from Chinese and international journalists with the indispensable assistance of an interpreter affiliated with the Ministry of Foreign Affairs. Previous studies have concluded that Chinese government-affiliated interpreters tend to use shifts (additions, omissions, and modifications) of interpersonal resources to “gatekeep” the journalists' questions or positively reconstruct the government's image represented by the premier's utterances (e.g., Li, 2018; Gu and Tipton, 2020; Li and Zhang, 2020). Nevertheless, these studies have largely overlooked how these shifts facilitate ensuring the successful negotiation of intersubjectivity at the PPCs, which is essentially related to the issues of ideology and power relations (for a detailed explanation of why this is the case, see Section 3.1).

There are many understandings of what the term intersubjectivity should cover. Many researchers have studied intersubjectivity in terms of the interpersonal function of the framework of systemic functional linguistics (SFL) by delving into how intersubjectivity is negotiated by the typical realization systems of the interpersonal function: pronouns (Ädel, 2012) and appraisal resources (Hyland, 2005). Following these studies, the current study also attempts to investigate how interpreting shifts of these interpersonal and appraisal resources facilitate the successful negotiation of intersubjectivity at the PPCs from 2016 to 2021. However, it will do so from the perspective of ideology and power relations which are inherent to the political discourse event. The methodology of corpus-based critical discourse analysis (CDA), which has become well received in this research agenda, will be deployed to interpret the corpus data.

## 2. Research background

Despite increasing academic awareness of the mediating role of the interpreter, there are only a few studies on the intersubjectivity in interpreted discourse. For instance, Janzen and Shaffer (2008, p. 352) argued that it is impossible for the interpreter's stance to be neutral in negotiating intersubjectivity between his or her clients because he or she is always making “subjective linguistic and framing choices,” whereas the interpreter tends to avoid over-intervention.

However, there have been a growing number of corpus-based CDA studies on Chinese-interpreted political discourse. Based on the corpus data of 15 PPCs, Wang and Feng (2018) concluded that the institutional interpreters' certain lexical choices echo the Chinese government's attitudes on a range of social and political issues. Gu (2018, 2019) explored the interpreter's agency at PPCs by focusing on the interpreters' application of present perfect structures and people-related items. The interpreters are found to align with the government's official positions and positively reconstruct the government's image. Incorporating SFL's framework, Li (2018), Fu and Chen (2019), and Gao and Wang (2021) discovered a high level of interpreter intervention through pronoun and modality shifts in Chinese diplomatic settings, especially additions of first-person plurals and shifts in modality values, which helps to broadcast the government's stances and preferred image to the world. Gao (2020), drawing upon the appraisal theory, probes the image reconstruction effects of appraisal shifts at China's World Economic Forum. Internationally, Munday (2015, 2018) also made use of the taxonomy of the appraisal theory to analyze interpreter mediation in employing shifts of attitudinal, engagement, and graduation items in political discourse in the West. Most recently, Gao and Munday (2022) operationalized the appraisal framework to reveal positive “us” and negative “them” in the discourse analysis of simultaneous interpreting.

In summary, although the majority of previous interpreting studies have significantly contributed to the research on interpreter intervention in interpreted political discourse, they have done so predominantly from the perspectives of stance, attitude, position, and image, among many others, which needs synthesis in the form of more encompassing terms such as ideology. Moreover, the social relations or power relations, which are discursively regulated between the government, media, and the people at the PPCs, have also been curiously underexplored. Relating the issues of ideology and power relations to intersubjectivity, this study will conduct a corpus-based CDA of recurrent shifts of SFL's typical interpersonal resources, which are interpersonal and appraisal resources, in order to identify how the ideological effects of these shifts ensure successful negotiation of intersubjectivity between interlocutors at the PPCs.

## 3. Systematic functional linguistics and appraisal theory

The SFL model distinguishes between three complementary strands of meaning or metafunctions: the ideational, the interpersonal, and the textual (Halliday and Matthiessen, 2014, p. 29–31). Ideational meaning construes the experience of the inner and outer worlds. Interpersonal meaning negotiates social relations in terms of power, solidarity, and evaluation, and textual meaning organizes ideational and interpersonal meanings in the language and other semiosis. The interpersonal function is typically realized through pronoun choice, modality, forms of address, and evaluative epithets.

The appraisal theory (Martin and White, 2005) focuses on evaluative epithets (hereinafter termed appraisal resources). Appraisal resources, used for “intersubjective positioning” (Martin and White, 2005, p. 95), allow the speaker to express a value judgment about an object or phenomenon, while the addressee is being positioned in relation to that judgment. In addition, the notions of judgment and positioning can be related to those of ideology and power relations. Appraisal resources are grouped into three domains by the appraisal theory (Table 1): attitude, engagement, and graduation (Martin and White, 2005, p. 34–38). The attitude system is further divided into subdomains of affect (the emotional), judgment (the ethical), and appreciation (the aesthetic) in which different degrees of positivity or negativity are encoded.

**Table 1:** Appraisal resources (based on Martin and White, 2005, p. 38).

**Domain**	**Subdomain**	**Value**	**Typical realizations**
Attitude	Affect	Feelings and emotional reactions	*Happy, satisfied, sad*
	Judgment	Of ethics, behavior, capacity	*Good, honest, weak*
	Appreciation	Of cultural and natural phenomena	*Beautiful, balanced, shallow*
Engagement	Monogloss	Single-voiced	Categorical assertion
	Heterogloss	Expansive	*Show, however, definitely*, pronouns, terms of address
	Contractive	*Claim, allege, possibly*
Graduation	Force	Raise	***Extremely** mad*
		Lower	***Slightly** mad*
Focus	Sharpen	*A **real** miracle*
		Soften	***Sort of** a miracle*

The engagement system consists of two subdomains: monogloss and heterogloss, depending on whether the discourse allows for perspectives and opinions other than its own, including expressions of modality (Narrog, 2012). The graduation domain deals with the gradability of evaluations, realized either through force (the raising or lowering of gradable items in terms of their amount or intensity) or focus (the sharpening or softening of non-gradable items based on their prototypicality of phenomena). The appraisal framework will be used for investigating appraisal shifts in the current data.

In the domain of engagement, modality is interpreted by Halliday and Matthiessen as the “intermediate degrees between the positive and negative poles” of a proposition or proposal (Halliday and Matthiessen, 2014, p. 147). Four dimensions are assigned to the modality system: modality type, orientation, modality value, and polarity (Halliday and Matthiessen, 2014, p. 146–150, 616–622). This study will make use of the first three dimensions to analyze modal shifts, focusing on modality type. There are four modality types: probability, usuality, obligation, and inclination. Probability (e.g., *possibly/probably/certainly*) and usuality (e.g., *sometimes/usually/always*) express the cline of possibilities between “it is so” and “it is not so” in a proposition; the scales of probability and usuality are referred to as modalization. Obligation (e.g., *must/need to/required to*) and inclination (e.g., *will/willing to/determined to*) express the cline of possibilities between “do it” and “do not do it” in a proposal; the scales of obligation and inclination are termed modulation. The analysis of modal shifts will focus on obligation and inclination in the current data.

In terms of pronoun shifts, SFL's transitivity systems will be drawn upon. Transitivity provides the linguistic resources for modeling change and construing “experience into a manageable set of process types” (Halliday and Matthiessen, 2014, p. 170). Six process types are identified as follows: material, mental, relational, existential, behavioral, and verbal processes. Material processes are the most fundamental. A change is a process unfolding in time involving certain participant(s); in transitive clauses describing material processes, the participants are an Actor and a Goal. The Actor brings about a change which is exerted on the Goal (Halliday and Matthiessen, 2014, p. 177–180). Material processes are further categorized into creative and transformative ones, depending on whether the Actor or Goal comes into existence as the process unfolds, or a pre-existing Actor or Goal is transformed in amount, quality, location, and many others, as the process unfolds (Halliday and Matthiessen, 2014, p. 184–189). The analysis of utterances involving pronoun shifts will focus on the material processes they describe. In the current investigation of interpersonal and appraisal resources in the PPCs, both systemic functional linguistics and appraisal theory are exploited for quantitative and qualitative analyses in Sections 5, 6.

## 4. Methodology

### 4.1. Corpus-based critical discourse analysis

This study will use corpus-based CDA to interpret the discourse material from the PPCs in terms of ideology and power relations. CDA (Fairclough et al., 2011) is particularly suitable for such an investigation because it regards discourse as a social practice, that is, both shaped by social structures and institutions and can facilitate either the reproduction or transformation of the social *status quo*. Thus, ideological discourse can help sustain unequal power relations between certain social groups through particular representations of reality (the word *reality* encompasses certain objects, events, and, more importantly, when it comes to power, social relations *per se*). In brief, CDA is interested in the inherent interplay between ideology, discourse, and power relations. Among the many different approaches of CDA, Fairclough's Dialectal–Relational Approach is especially suitable for the current study because it focuses on political discourse and often incorporates the SFL framework, which sees language as shaped by the social functions it serves (Wodak and Meyer, 2009).

Critical discourse analysis can become more convincing when it incorporates corpus methods and large-sized data, given the criticism of CDA that it often selects only a limited number of text fragments for analyses (Chilton, 2005). Corpus-based CDA has become increasingly more favored in studies over the past decade, as the researcher's subjectivity can be mitigated by massive corpus data. As Baker et al. (2008) noted, corpus-based CDA expounds on quantitative results along the lines of existing theories and allows qualitative results to be quantified at the same time. Thus, corpus-based CDA will be used for the following investigation.

### 4.2. Data and procedures for analysis

The Chinese-English corpus data are comprised of transcripts of all questions and answers and their interpretation from six PPCs (2016–2021), the videos of which can be found on websites such as iQiyi.com. Zhang Lu interpreted for Premier Li Keqiang at all six PPCs. Corrections are carefully made to the transcripts on the government's official websites, which have been more or less edited so that the resulting transcripts are verbatim. Punctuation is assigned to them based on semantic units.

The corpus (textual transcription) consists of 109,060 tokens (one English word or one Chinese character is counted as one token), including the Source Text (ST) subcorpus (64,463 tokens) and Target Text (TT) subcorpus (44,597 tokens). The journalists' questions and their interpretations will be used for investigating appraisal shifts, while Premier Li's utterances and their interpretations will be used for interpersonal shifts. Quantitative analysis will be followed by qualitative analysis. For quantitative analysis, the data are analyzed using AntConc (version 4.0.5 windows), a corpus linguistics software containing a variety of corpus tools for generating concordance lines, collocations, clusters, and many others. Careful ST–TT comparisons will also be made when necessary, and the criteria for doing so will be articulated. The qualitative analysis will be conducted along the lines of CDA, focusing on the perspectives of ideology and power relations. In terms of the interpretation of concrete examples from the corpus, this study will mainly draw upon Fairclough's (2013) CDA model consisting of three parts: description of the text, interpretation of the discourse process, and explanation of the sociopolitical context. Finally, quantitative and qualitative findings will be discussed together to present the conclusion.

## 5. Quantitative findings

Following Gao and Munday (2022), the appraisal expressions, including the appraisal shifts (Munday, 2018) in the data, were annotated manually due to the fact that the corpus software alone cannot identify the evaluative meaning. In particular, the appraisal theory (Martin and White, 2005) was exploited as the linguistic framework to annotate the evaluative meaning of positivity and negativity.

### 5.1. Appraisal shifts

Overall, patterns of appraisal shifts in the interpretation of the journalists' questions are displayed in Table 2. Only appraisal shifts that are judged to be directly prompted by ideological issues or deviate from the original evaluation are counted; a careful comparison between the source text (ST) and target text (TT) is made. In this section, ST refers to the journalists' questions and TT refers to the interpretation of the questions. Since shifts of items belonging to different appraisal domains are often juxtaposed in the TT and since a rigorous grouping of them by these domains can involve greater researcher's subjectivity, the shifts are classified according to whether they enhance or soften the positivity or negativity toward the Chinese government.

**Table 2:** Appraisal shifts in the TT.

**Type of shift**	+ positivity^*^	**– positivity**	**+ negativity**	**– negativity**	**Total**
Addition	14	0	2	17	33
Omission	1	1	4	18	24
Modification	16	0	2	36	54
Total	31	1	8	71	111
Proportion	27.9%	0.9%	7.2%	64.0%	100%

^*^ “+” means enhancing and “–” means softening. The numbers are frequencies of shifts.

Results show that 91.9% of appraisal shifts have the effects of enhancing positivity and softening negativity in the questions about government actions. The number of negativity-softening shifts (71) is more than two times that of positivity-enhancing shifts (31), which suggests the interpreter is more liable to tone down the journalist's negative evaluation of the government, rather than scale up his or her positive evaluation of it. Regarding negativity-softening shifts, modifications (36) are significantly more than additions (17) or omissions (18), perhaps because it takes less effort for the interpreter to fine-tune the journalist's negative wording than omit it or add what is not said. To enhance positivity, the interpreter is more likely to add or modify items (30 additions and modifications vs. one omission). The figures show the interpreter is inclined to use appraisal shifts which enhance the positivity or soften the negativity in the questions.

Curiously, there are eight instances of enhanced negativity. Nonetheless, it is found that such negative effects are of minor importance and hardly perceptible after ST–TT comparison. For example, omitting the engagement phrase 我们也注意到 (we have noticed that) turns the disputable perception of a social issue into a recognized fact. In any case, these shifts are insignificant compared to those enhancing positivity and softening negativity.

### 5.2. Interpersonal shifts

Table 3 shows the overall patterns of shifts to *we, our*, and *us* (hereinafter collectively termed the broader WE) in the interpretation of Premier Li's utterances. The one-to-many nature of the PPC means the use of *we* is “stable and homogeneous, referring predominantly to the Chinese government” (Gu and Tipton, 2020). It is, thus, necessary to exclude the rare instances in which *we* refer to both the Chinese government and, to illustrate, the US government. In this section, ST refers to the Premier's utterances, while TT refers to the interpretation of those utterances.

**Table 3:** Frequencies of WE in the ST and TT.

**Item(s)**	**Frequency in the ST/TT**	**Frequency per year**
我们	652	108.7
The broader WE	1,181 (we 764; our 366; us 51)	196.8
Increase rate	81.1%	

Results show that a significant share of the broader WE in the TT is additions. There are 652 instances of 我们 (we/our/us) in the ST, and 1,181 instances of the broader WE in the TT, representing a dramatic increase of 81.1%. The frequency per year rises from 108.7 to 196.8 at the same time. Admittedly, the sharp increase can be attributed to the fact that the subject is often omitted and implied in the Chinese language, which is not the case for English. There are, however, many instances in which the interpreter uses the passive voice to translate the Premier's null subject utterances, which suggests there is freedom of choice (Yu and Wu, 2020) on the part of the interpreter regarding the addition of WE. Moreover, even if the interpreter's adding WE results from the need for less cognitive effort in consecutive interpreting, such proliferated additions of WE do have prominent discursive effects from a “product-oriented” (Gu and Tipton, 2020) perspective.

To narrow down the scope of the investigation of shifts to the broader WE, this section will go on to look at shifts to “we have (done/been doing)” and “we will,” the two most frequent (87 and 175, respectively) collocations containing *we* in the TT. The results after careful ST–TT comparison are shown in Tables 4, 5. “Literal translation” in the tables means no shift in item or structure.

**Table 4:** Frequencies of shifts to “we have (done/been doing).”

**Construction in TT**	**Item/structure added/modified in TT**	**Frequency**	**Proportion**	**Total proportion**
We have (done/been doing)	*We*	6	6.9%	70.1%
	*Have (done/been doing)*	31	35.6%	
	*We have (done/been doing)*	24	27.6%	
	Literal translation	26	29.9%	29.9%
	Total	87	100%	100%

**Table 5:** Frequencies of shifts to “we will.”

**Shift in TT**	**Shift type**	**Frequency**	**Proportion**	**Total proportion**
Addition of *we will*	Add *we*	13	7.4%	44.6%
	Add *will*	19	10.9%	
	Add *we will*	46	26.3%	
Modification to *we will*	Modify a modal to *will*	21	12.0%	33.7%
	Add *we* and modify a modal to *will*	38	21.7%	
Literal translation	/	38	21.7%	21.7%
Total	/	175	100%	100%

Concerning “we have (done/been doing)” for deciding whether a Chinese expression is considered natural to be translated into the present perfect (continuous) structure in English, this study follows the criteria articulated in Gu (2018). Put simply, Chinese expressions with “the verb + *le*” construction or explicit markers such as 去年 (last year) are largely considered natural to be translated into English using the past tense, and expressions with explicit markers such as 近年来 (over recent years) and 已经 (already) are considered natural to be translated into English using the present perfect (continuous) structure. With rare exceptions, the contexts are taken into account, and decisions are made carefully.

Concerning “we will” for deciding whether a Chinese modal naturally translates as *will* in English, this study follows the Chinese-English scale of modality value advanced by Li (2018), which is based on the modality scales of Halliday and Matthiessen (2014, p. 149–150). The Chinese items 将 and 会 are largely translated into English as *will*. 要 is trickier since it can mean any of the three English modals: *need to, will*, and *should*. A careful ST–TT comparison with contexts taken into account is made, and it is found that most instances of 要 in the ST primarily mean *need to*. Any translation of a Chinese modal which is not 将 and 会 (and thus does not mean *will*) in the ST as will in the TT, and any translation of the instances of 要 meaning need to or should in the ST as *will* in the TT, is regarded as a modification.

Table 4 shows that 61 out of 87 instances of “we have (done/been doing)” in the TT involve shifts of some kind (70.1%), whether with the addition of only *we* or the modification to the present perfect (continuous) structure or both. On the other hand, Table 5 demonstrates that almost half (44.6%) of “we will” in the TT are added, entirely or partly. It is shown that 33.7% of “we will” are modified in one way or another, and only 21.7% are literal translations. In summary, the figures reveal a strong tendency on the part of the interpreter to use “we have (done/been doing)” and “we will,” even when there is no direct trigger in the ST.

### 5.3. Summary of quantitative findings

To summarize the quantitative findings in this chapter, results show that the interpreter is strongly inclined to employ appraisal shifts, which enhance positive evaluations and soften negative evaluations in the journalists' questions about the Chinese government. She also tends to use pronoun and modal shifts in interpreting Premier Li's answers, particularly additions of the broader WE and shifts to the collocations of “we have (done/been doing)” and “we will”.

The results reveal a high level of interpreter intervention at the PPCs. This is perhaps because the interpreter is officially affiliated with the Ministry of Foreign Affairs, which confers on her the power to make changes to the ideological discourse of other social groups (the media in this instance) for the government's needs.

## 6. Qualitative findings

This section concerns a qualitative analysis of appraisal shifts in the journalists' questions and interpersonal shifts in the Premier's answers, concerning their implications for the Chinese government's official ideology and the existing power relations between the government, media, and the Chinese people. To that end, three examples of appraisal shifts and three examples of interpersonal shifts from the corpus are interpreted along the lines of CDA. In particular, the official ideological notions that are made manifest by interpersonal shifts (the notions of solidarity, change, and resolution) will be discussed in detail before examples are given.

### 6.1. Appraisal shifts

In this section, examples of appraisal shifts are interpreted in terms of the domains and subdomains of appraisal theory to delve further into how appraisal shifts act on competing ideologies and power relations to ensure the successful negotiation of intersubjectivity at the PPCs. For comparison, literal translation (LT) is provided by the authors for each example. Examples 1 and 2 illustrate how shifts of items with attitudinal and graduational meanings can soften the negative evaluation or enhance the positive evaluation of the Chinese government in the journalist's question, respectively. Example 3 demonstrates how shifts of items with engagement and graduational meanings can soften the negativity in the question.

Example 1 (2020)

ST (*China Daily*): 今年您在政府工作报告中调低了城镇新增就业目标,  调高了调查失业率。面对严峻的就业形势,  请问总理,  今年政府将如何遏制失业潮？

LT: In this year's Government Work Report, you revised downward the target for new urban jobs and revised upward the projected surveyed unemployment rate. Mr. Premier, facing a severe employment situation, what will the government do to contain the wave of job losses?

TT (interpreter): In this year's Government Work Report, the target of new urban jobs has been revised downward, and the target of surveyed unemployment rate somewhat upward, both compared with the levels last year. We face a severe employment situation. What will the government do to avert massive job losses?

This is a question from *China Daily* about unemployment in the wake of the COVID-19 pandemic in 2020 when the PPC was postponed to May instead of March. The journalist's use of *contain* presupposes a situation that already exists, *viz*. massive unemployment, but the interpreter uses *avert* to indicate that it does not and will not happen. Security/insecurity is a variable of the affect subdomain of attitude (Martin and White, 2005, p. 50); given the inherent gradations of attitudinal lexis, *avert* can invoke higher security than *contain* in the mind of the audience in this example. In addition, the interpreter adds *somewhat*, a force-lowering graduation item, in talking about the projected unemployment rate. These shifts imply that job losses are not as severe as the journalist has suggested and that the government can bring them under control, stressing the government's competence in ensuring people's livelihoods. As a result, the negativity in the question has been toned down.

Example 2 (2020)

ST (*Asahi Shimbun*): 数次控制住疫情的中国……

LT: China has brought the pandemic under control several times…

TT (interpreter): China has been successful in bringing the spread of the virus under control in a short span of time.

Here, the Japanese journalist utters this sentence before inquiring about China's plans for economic cooperation with Japan. Capacity is a variable of the judgment subdomain of attitude (Martin and White, 2005, p. 53). Instead of faithfully rendering the neutral, if not slightly laudatory, utterance, the interpreter adds the judgmental item *successful*, which explicitly compliments the government on its capacity for coping with the pandemic. More interestingly, the phrase *in a short span of time* is added without any direct trigger in the ST. In particular “*short*,” a force-lowering graduation item, stresses the government's efficiency in addressing the pandemic. These shifts effectively enhance the positive evaluation of the Chinese government.

Example 3 (2017)

ST (Groupe RFI of France): The European Union is China's second-largest commercial partner with a trade deficit of 137 billion euros in favor of China and a large number of European businesses complain about that.

TT (Interpreter): 欧盟是中国第二大贸易伙伴,  但欧盟对华贸易赤字高达1370亿欧元。这是欧盟的统计数据。所以欧盟一些企业对此颇有微词。

BT: The European Union is China's second-largest trading partner, but the EU's trade deficit against China is as high as 137 billion euros. The statistics are from the EU. So some EU businesses are complaining somewhat about that.

The BT is short for back-translation. This is a negative question about China's trading practices with the EU. Interestingly, the interpreter adds an entire sentence: *The statistics are from the EU*. According to the appraisal theory, this utterance and the sentence *EU's trade deficit against China is as high as 137 billion euros* are both monoglossic because they are categorical assertions. Viewed separately, they both make no recognition for alternative positions. However, when they are considered together, the added utterance forms an explicit challenge to the position expressed by the journalists' utterances. The interpreter is attempting to make listeners cast doubt on the journalist's position by making it sound biased and possibly untrue. In addition, there are shifts in graduational items. *A large number of* is weakened by 一些 (*some*), and *complain* is translated as 颇有微词 (roughly *complain somewhat*), lowering the force of the negative appraisal in the question. These shifts tone down negativity by making the journalist's proposition sound biased and indicating that the situation is less severe than has been suggested.

To summarize, the interpreter, in altering appraisal resources, effectively alters how the questions represent reality and makes them conform to the government's official ideology. In other words, appraisal shifts *de facto* reproduce official ideology. The existing power relations between the government and media, *viz*. the former's authority over the latter, are reproduced at the same time. That authority is manifest in the fact that the government-affiliated interpreter can reframe the questions to suit the government's needs, and that the journalists cannot challenge these alterations, and that the questions are said to be prescreened backstage (Gu, 2019), to name just a few.

### 6.2. Interpersonal shifts

This section is organized around the ideological notions that are made manifest by interpersonal shifts rather than around shifts in pronouns and modals *per se*. For each notion, a general analysis is followed by a concrete example.

#### 6.2.1. The notion of solidarity

*We* and *our* have many implications for the positioning of the political speaker in relation to his or her addressee (van Dijk, 1997). Respectively, pointing to the vertical and horizontal dimensions of interpersonal relations, power (or authority) and solidarity form the opposite ends of a continuum along which relations between social groups can be dynamically regulated *via*, for example, discourse. Pronouns, therefore, are worthy of investigation.

With pronouns, observe Halliday and Matthiessen (2014, p. 325), “the referent is defined interpersonally, by the speech situation.” Shifting uses of *we* are found in both the ST and TT, where the “inclusive” *we* refer to the Chinese government and the Chinese people, and the “exclusive” *we* refer only to the former. The Premier uses them ambiguously, supposedly on the presumption that he is “not only speaking on behalf of the government but also on behalf of the audience” (Wales, 1996, p. 62). This political discourse strategy has also been observed in the discourse of the UK and the US political leaders, suggesting an increasing awareness among politicians of the ideological effects of *we* (c.f. Fairclough et al., 2011; Kazemian and Hashemi, 2014).

Therefore, the use of *we* at the PPCs refers to the Chinese people as well. To choose the all-encompassing *we* over the “dehumanizing” *China* or the people-excluding *government* is to create a stronger sense of solidarity between the government and the people by appealing directly to the people and identifying the government with the people. The reason for doing so can be traced back to the official ideology, which stresses solidarity between the Communist Party of China (CPC) and the people, and according to which the government claims itself as a “people's government.” This echoes the traditional Confucian principles of harmony between the ruler and the ruled and people-oriented governance; allusions to the traditional culture contribute to the legitimacy of that ideology.

Crucially, only *via* the interpreter can international journalists (and audiences) understand the Premier and judge his positions. On this occasion, the sense of solidarity, which is enhanced by the addition of *we* is not only perceived by the Chinese people (“We are together”) but also perceived by the international journalists (“They are together”). The use of *we* represents, at the same time, an exhibition of solidarity, an attempt at persuasion. The interpreter in adding *we* effectively distances the journalists from the Premier by pitting the explicit *we* against an implicit *them*, making the government sound more confident because it supposedly has the people's consent.

In brief, the use of *we* effectively reproduces the official ideological notions of solidarity and people-oriented governance. Moreover, the existing power relation of government–people solidarity is reproduced, at least discursively. Here, the effect of reproducing ideology and the effect of reproducing power relations “converge” because the ideological notion being reproduced, viz. solidarity, is itself a relation of power in actuality.

That is not the only power relation being reproduced. The possessive *our* organizes power relations differently in comparison with *we*. The most frequent (54) collocation containing *our* in the TT, “our people,” is a good case in point (notably, every *our* before *people* in the TT is added without direct triggers in the ST). Now, while *we* construct a community with no clear borders between the government and the people, the *our* in “our people” can be regarded as connoting the government's “possession” of, *viz*. authority over, the people (of which the term possessive is sufficiently suggestive). Ostensibly aligning the people with the government on an equal footing, “our people” actually points to an uneven distribution of powers, the most pertinent one being the power to use discourse and discourse events (such as the PPC) for the reproduction of powers themselves.

Conversely, *our* in “our people” can also be regarded as connoting the government's duties toward the people it rules (one is responsible for something/someone he or she possesses or has authority over, either de jure or morally). The power distance between the government and the people is shortened by the second connotation, not the first. The government ultimately relies on the people for consent and legitimacy. In other words, the notions of solidarity and people-based governance are reproduced, and the sense of solidarity is relatively enhanced.

In summary, additions of *we* and *our* in the TT result in the reproduction of official ideology (mainly the notions of solidarity and people-based governance) and the reproduction of existing power relations between the government and people, both solidarity and authority.

Example 4 (2021)

ST (Premier Li): 广大人民共克时艰,  最后是实现了城镇新增就业1186万.

LT: The people at large overcame the hardships of the time together and eventually realized an addition of 11.86 million urban jobs.

TT (interpreter): Our people faced the difficulty with strong solidarity. For the whole year, we achieved 11.86 million new urban jobs.

Here, Premier Li is addressing the hard-won economic growth amid the pandemic. On the discourse level, his utterance explicitly stresses the role played by the people in making socioeconomic accomplishments and does not mention the government at all. Nevertheless, in adding *we, our*, and *strong solidarity*, the interpreter relates the people to the government and reminds the listeners of the government's role in ensuring economic growth, social stability, and people's livelihoods.

#### 6.2.2. The notion of change

“We have (done/been doing)” and “we will” are worthy of investigation not just because of the pronoun but because of the actions they describe on the sentence level. It is *we* who bring about the *changes*. Indeed, one of the fundamental ideological notions of the Chinese government (and CPC, given the one-party rule in mainland China) other than that of solidarity is the notion of change. Mao Zedong, in his Marxist-Leninist dialectical materialism, believed that the development and resolution of internal contradictions in society would culminate in qualitative social changes (Gurley, 1970). When 翻天覆地的变化 (sweeping changes) was searched on the state-owned *People's Daily Online* website, precise matching results showed 11,138 articles (as of May 14, 2022).

According to SFL's transitivity system, which distinguishes between six process types and following the procedures for analysis in Yu and Wu (2020), this study shows that 81.3% of clauses of “we have (done/been doing)” and “we will” in the TT describe the material processes of creation and transformation. They represent material and social changes in the past and future, largely concerning economic growth, employment, poverty reduction, healthcare, pension scheme, diplomatic ties, and many others, all of which boils down to people's wellbeing. This is reasonable because the priority in CPC's ideological strategy to maintain social unity has long shifted from a class struggle toward economic development and modernization (Lee, 2019) while putting more emphasis on the people-oriented principle by interpreting it in terms of improvement in people's quality of life. The official ideology is reproduced through these utterances of change. In addition, the ideological notions of solidarity and change converge on the notion of people's wellbeing.

In addition, the addition of *we* in such clauses has discursive effects as well. On the one hand, it makes explicit and perpetuates the main actor, *viz*. the government, behind positive social changes in the past, present, and future. On the other hand, it strengthens the government–people solidarity because the referents are ambiguous, including the people from time to time. For the tasks and goals laid out by the Premier to be fulfilled, the efforts of the government and the people are equally indispensable. Consequently, both the government's authority over the people and the solidarity between them are reproduced here.

Example 5 (2016)

ST (Premier Li): 我们不是靠“大水漫灌”的强刺激来获得的,  而是推动产业、消费升级,  使经济结构有新局面。通过加快新旧动能转变,  实现转换,  实现新的势头。更重要的是带动了5000多万人的新增劳动力就业。

LT: We did not achieve it by massive strong stimulus measures but by upgrading industries and consumption and by creating new patterns in the economic structure. By driving the transformation from old to new drivers of growth, a transition, a new kind of momentum is realized. More importantly, it created over 50 million new jobs.

TT (interpreter): We have achieved this without resorting to massive stimulus measures that would have an economy-wide impact. Rather, we have been boosting the upgrading of Chinese industries and consumer patterns, which has contributed to the improvement of China's economic structure. We have encouraged and enhanced the growth of new drivers for economic development and renovated the traditional drivers of growth. And moreover, we have generated as many as 50 million new urban jobs.

Here, Premier Li is addressing China's ongoing industrial and economic transformation. While there is only one mention of 我们 (we) in the ST and all of the expressions of actions should be translated using gerunds or the past tense, the interpreter nevertheless uses five “(we) have (done/been doing).” Notably, they form an instance of parallelism, regarded by van Dijk (1997) as a political discourse strategy to “draw attention to preferred meanings.” Hence, these utterances, describing creative and transformative processes in the first place, are made to emphasize the change-effecting actor (mainly the government in this example) and the positive influence the actions have on the present.

#### 6.2.3. The notion of resolution

One question remains to be answered concerning the “we will” description of socioeconomic changes in the future. In the interpretation of Premier Li's utterances, instances of “we will” (175) significantly outnumber those of “we must” (61) or “we need to” (30). This goes against the findings of Gao and Wang (2021) who found more shifts to “we must/need to” than shifts to “we will” in the interpretation of 17 diplomatic speeches delivered by Chinese government officials.

As introduced earlier, Halliday and Matthiessen (2014, p. 146–150) assigned four dimensions to modality: modality type, orientation, modality value, and polarity. Polarity concerns positive and negative forms of modality, and since instances of “we will” are predominant in the positive against the negative (frequencies: 175 vs. 6), polarity is not discussed here. In terms of orientation, both “we will” and “we must/need to” are explicit and subjective because “the source of conviction” (Halliday and Matthiessen, 2014, p. 149) is explicitly referred to as the subjective *we*. Both collocations intend to persuade the listeners of the speaker's conviction that the action will be implemented.

What matters are the remaining two dimensions, modality type and modality value. While *will* expresses the inclination to do something with a median value, *must* and *need to* express the obligation to do something with high and median-high values, respectively. If the interpreter prefers *will* to *must* or *need to*, her reason for doing so should lie in the superiority of inclination over obligation since *will* is lower in modality value. Indeed, there is a perception that the “we will” speaker will be construed as *inclined* or willing to do something, whereas the “we must/need to” speaker will be construed as only *obliged* to do something, without necessarily wanting to do it.

Martin and White (2005, p. 53–55) make an analogy between the judgment subdomain of appraisal theory and SFL's modality types. The judgment subdomain consists of five variables, two of which are tenacity and propriety. Tenacity concerns how resolved and socially reliable a person is (how tenacious?), while propriety concerns how deserving of moral condemnation a person is (how proper?). Tenacity or resolution is an “individualized” and spontaneous virtue, independent of external forces such as social conventions. Nonetheless, propriety or decency *can* be regarded as merely conforming to social and legal conventions. Crucially, tenacity and propriety correspond to SFL's modality types of inclination and obligation, respectively. Modulations of inclination (*We will do…*) can be related to lexicalized tenacity (*We're* ***resolved/determined*** *to do doing…*). Similarly, modulations of obligation (*We must/need to do…*) can be related to lexicalized propriety (*It'd be* ***good/moral*** *of us to do… It'd be* ***corrupt/selfish*** *of us not to do…*).

In other words, while the “we must/need to” speaker is construed as possibly mindful of moral condemnation in making a promise of action, the “we will” speaker is construed as transcending community morality altogether and focusing directly and solely on fulfilling the promise of action. Put simply, the “we will” speaker is construed as having more determination and resolution. In actuality, the notion of resolution is also important in the government's official ideology. When 坚定决心 (literally “determined resolution”) was searched on *People's Daily Online*, precise matching results showed 10,615 articles (as of May 14, 2022).

In summary, “we will” at the PPCs allows Premier Li to sound more determined and resolved than “we must/need to” and implies a bigger likelihood of the positive socioeconomic changes coming true. The government is construed as having more resolution and being more action-oriented. The ideological notion of resolution is reproduced, and the relation of government–people solidarity is reproduced as well. This is aptly illustrated in Example 6.

Example 6 (2019)

ST (Premier): 那么还要加强对知识产权的保护。我们建议要修改知识产权保护法,  引入惩罚性的赔偿机制。发现一起就要处理一起。 LT: The protection of intellectual property rights also needs improving. We suggest that the law on intellectual property rights protection be revised and a mechanism of punitive compensation be introduced. Any identified case [of infringement] needs to be dealt with.

TT (interpreter): We will also enhance the protection of intellectual property. In this respect, we will make revisions to the relative laws on intellectual property protection. We will introduce a mechanism of punitive compensation to ensure that all infringements of intellectual property will be seriously dealt with.

Here, Premier Li is addressing the issue of protection of intellectual property, stressing China's effort to improve law and justice. The use of 要 in the ST means the obligational *need to*, but it is translated as the inclinational *will* by the interpreter. On top of that, she adds *we* and *seriously* in the interpretation. The government is, thus, made to appear more resolved and action-oriented in improving the rule of law. The ideological notions of resolution and people's wellbeing are being reproduced, as improvement in the rule of law will eventually benefit people's lives.

In the examples mentioned earlier, results show the interpreter either (1) alters appraisal resources in the journalists' questions according to the Chinese government's ideology so that positive and negative evaluations of the government are enhanced and softened, respectively, and that the government's authority over the media is reproduced or (2) uses shifts to first-person plurals and inclinational modal verbs in the Premier's utterances so that official ideological notions of solidarity, change, resolution, and people's wellbeing are stressed, and that both the government's authority over the people and the government–people solidarity are reproduced.

## 7. Discussion and conclusion

In the final analysis, the effect of interpersonal shifts is to reproduce the government's official ideology and the existing power relations between the government, media, and the Chinese people. This is done either indirectly (by reframing journalists' questions) or directly (by “improving” the Premier's answers). Research on the CDA shows that ideological discourse can help to reproduce the social *status quo*. In the current investigation, based on the qualitative results of the study, the reproduction of the social *status quo* can be regarded as the result of the mutual reproduction between the official ideology and power relations.

On the one hand, according to Althusser's Marxist argument (Althusser, 2014, p. 92–93), the primary function of a society's dominant ideology (which belongs to the society's superstructure) is to ensure the reproduction of existing relations of production (which belongs to a society's economic base), which are essentially power relations seen from the perspective of production. On the other hand, since these relations ultimately play a determinant role in the makeup of society, their reproduction will, in reverse, ensure the reproduction of the dominant ideology. Thus, the interplay between the dominant ideology (equivalent to the official ideology in this study) and existing power relations at the PPCs helps to ultimately reproduce the social *status quo*.

Thus, it is by ultimately reproducing the social *status quo* that interpersonal shifts ensure successful negotiation of intersubjectivity at the PPCs because every conflict (potential or actual, in ideology or reality) which may hinder that negotiation is circumvented or downplayed by these shifts. Conclusively, the interpreter is found to be strongly inclined to use ideologically charged interpersonal shifts in her interpretation of the questions and answers at the PPCs, which ensures successful negotiation of intersubjectivity by reproducing the official ideology and existing power relations and, ultimately, the social *status quo*.

Major contributions of the current study include the use of the latest interpreting data from the PPCs (2016–2021), a comprehensive examination of shifts in the typical realization systems of SFL's interpersonal and appraisal resources, and an interpretation of these shifts in terms of the reproduction of official ideology and existing power relations. However, owing to its limited scope, this study only analyzes the intersubjective and ideological effects of interpreting shifts in Chinese-English diplomatic settings. It is expected that contrastive studies will be made in the future on whether and to what extent interpreters for political events in, for example, the European Union use shifts for ideological reproduction and negotiation of intersubjectivity since there is no “official ideology” within the multinational EU, at least not ostensibly so.

## Data availability statement

The original contributions presented in the study are included in the article/supplementary material, further inquiries can be directed to the corresponding authors.

## Author contributions

JX and YL conceived and designed the data collection, analyzed the data, wrote, and proofread the manuscript. All authors contributed to the article and approved the submitted version.
